# Gamma Knife radiosurgery for vestibular schwannoma: case report and review of the literature

**DOI:** 10.1186/1477-7819-7-100

**Published:** 2009-12-18

**Authors:** Benjamin J Arthurs, Wayne T Lamoreaux, Neil A Giddings, Robert K Fairbanks, Alexander R Mackay, John J Demakas, Barton S Cooke, Christopher M Lee

**Affiliations:** 1 Gamma Knife of Spokane, 910 W 5th Ave, Suite 102, Spokane, WA 99204, USA; 2 University of Washington School of Medicine, Seattle, WA, USA; 3 Cancer Care Northwest, 910 W 5th Ave, Suite 102, Spokane, WA 99204, USA; 4 Spokane Ear Nose & Throat Clinic, 217 W Cataldo Ave, Spokane, WA 99201, USA; 5 Mackay & Meyer MDs, 711 S Cowley St, Suite 210, Spokane, WA 99202, USA; 6 Spokane Brain & Spine, 801 W 5th Ave, Suite 210, Spokane, WA 99204, USA

## Abstract

Vestibular schwannomas, also called acoustic neuromas, are benign tumors of the vestibulocochlear nerve. Patients with these tumours almost always present with signs of hearing loss, and many also experience tinnitus, vertigo, and equilibrium problems. Following diagnosis with contrast enhanced MRI, patients may choose to observe the tumour with subsequent scans or seek active treatment in the form of microsurgery, radiosurgery, or radiotherapy. Unfortunately, definitive guidelines for treating vestibular schwannomas are lacking, because of insufficient evidence comparing the outcomes of therapeutic modalities.

We present a contemporary case report, describing the finding of a vestibular schwannoma in a patient who presented with dizziness and a "clicking" sensation in the ear, but no hearing deficit. Audible clicking is a symptom that, to our knowledge, has not been associated with vestibular schwannoma in the literature. We discuss the diagnosis and patient's decision-making process, which led to treatment with Gamma Knife radiosurgery. Treatment resulted in an excellent radiographic response and complete hearing preservation. This case highlights an atypical presentation of vestibular schwannoma, associated with audible "clicks" and normal hearing. We also provide a concise review of the available literature on modern vestibular schwannoma treatment, which may be useful in guiding treatment decisions.

## Background

Vestibular schwannomas (VS), or acoustic neuromas, are benign neoplasms of the myelin-forming Schwann cells of the vestibulocochlear nerve. They arise commonly within the internal auditory meatus, and may extend into the cerebellopontine angle. Reported incidence is 1 per 100,000 person-years and typical presentation occurs in the 5^th ^or 6^th ^decade of life[1]. Symptoms are related to dysfunction of the vestibulocochlear nerve or anatomically related structures. Of patients diagnosed with VS, 95% have ipsilateral hearing loss[2]. A significant fraction will also experience tinnitus, vertigo, or disequilibrium; facial or trigeminal neuropathy may occur with larger tumours[2].

The indicated treatment for VS may depend on the patient's symptoms, tumour size, growth rate, age, and life expectancy. Management choices include conservative observation or treatment with stereotactic radiosurgery, fractionated radiotherapy, and microsurgery. Therapeutic success may be measured by tumour control, commonly referred to as cessation of growth or a reduction in tumour size[3,4]. As interventions have become less invasive, mortality has been nearly eliminated and morbidity rates have been significantly reduced[4,5]. Therefore, current therapeutic goals for many patients and physicians include tumour control and patient-oriented outcomes, including preserving hearing and facial nerve function.

Unfortunately, there have been no double-blind randomized trials to compare efficacies of treatment modalities, and prospective comparisons are few[6,7]. Meta-analyses of existing retrospective data remain the best available evidence, but inconsistencies in the way variables are reported make it difficult to compare outcomes using this data[3,8,9]. Therefore, treatment decisions cannot be based solely on best evidence and involve significant subjective input from physicians and patient experience[10].

In this case report, we summarize a course in contemporary vestibular schwannoma management, involving a patient presenting with no hearing loss and a sensation of "clicking" in the ear. A small fraction of patients present without hearing loss, as described above, and many more experience audible sensations in the form of tinnitus; however, to our knowledge, the associated symptom of "clicking" has not been documented previously. We describe the decision-making process that led the patient to seek radiosurgical intervention, and the treatment outcomes over 45 months of follow-up. Additionally, we provide a brief review of the literature describing the efficacy of stereotactic radiosurgery as compared to the alternative modalities, which may be useful for informing patients and guiding treatment decisions.

## Case Presentation

### Case Report

This case involved a 63-year-old woman with hypertension and hypothyroidism, who presented to her primary care physician with a complaint of worsening "clicking" in her left ear. Accompanying symptoms included dizziness and light-headedness. The "clicks" had been heard for several months and were thought to originate from the Eustachian tube. The dizziness had been present for several weeks and was consistent with vertigo, involving "whirling sensations" that were exacerbated by movement. Treatment of the vertigo with meclizine was unsuccessful. There were no complaints of decreased hearing, headache, or other neurological deficits. The patient was advised to follow up with a carotid duplex study and brain MRI.

Findings on the axial, contrast enhanced MRI were positive for a 18 × 14 × 13 mm enhancing mass at the left cerebellopontine angle. The lesion projected from the internal acoustic meatus, with a small intracanalicular tail. Slight compression of the pons, medulla, and left cerebellar hemisphere was noted. The presentation was radiographically consistent with an extracanalicular vestibular schwannoma, although there was the outlying possibility it was a meningioma. Referral was made to a neuro-otologist, and subsequent evaluation revealed normal cranial nerve functions, no nystagmus, and a normal Romberg test. Audiograms exhibited normal speech reception threshold, below 20 dB in both ears, and 100% speech discrimination scores bilaterally. The absence of symptoms related to cochlear nerve function is infrequently encountered with vestibular schwannomas, with fewer than 5% of patients presenting without hearing impairment[2]. Cardiovascular exam was normal, with no abnormalities of jugular venous pressure or carotid pulse bilaterally.

Based on the images, the patient was diagnosed with vestibular schwannoma and counselled on the treatment options; conservative observation by serial MRIs, surgical removal, stereotactic radiotherapy, and gamma knife radiosurgery. Taking into account her age and health status, her neuro-otologist determined that suboccipital craniotomy was a treatment option with the potential for hearing preservation. The patient was informed of the risks associated with surgery including hearing loss, CSF leak, meningitis, facial nerve damage, and other complications. A radiation oncologist discussed the risks and benefits of Gamma Knife treatment, including reported recurrence rates and hearing preservation rates. Conservative management was discussed with several doctors, and one neurosurgeon recommended against it, given that the patient's primary motivation was hearing preservation. The patient also sought opinions from her primary care provider and an additional neurosurgeon.

**Figure 1:**
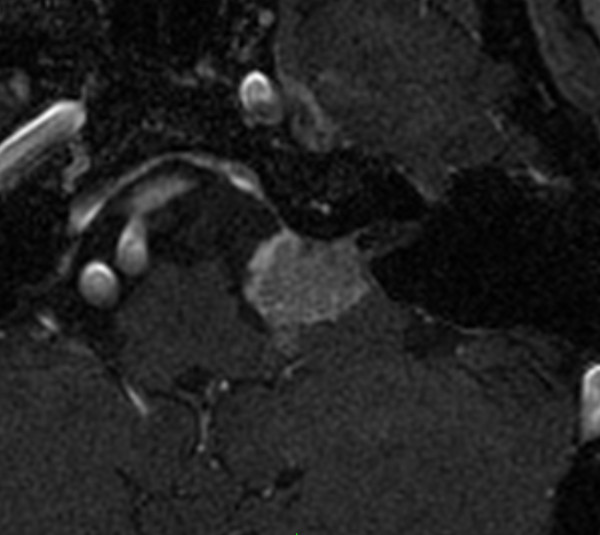
**Pre-treatment MRI**. Enhanced T1-axial MRI of the brain prior to gamma knife radiosurgery showing an enhancing lesion extending into the cerebellopontine angle from the left internal acoustic meatus.

After 4 months, meeting with various physicians and weighing the treatment options, the patient elected to pursue stereotactic radiosurgery. The preoperative gadolinium enhanced T1-MRI, required for Gamma Knife treatment planning, revealed the mass extending from the left acoustic meatus showing no significant expansion from the previous study (see Figure 1). The tumour received a dose of 13 Gy prescribed to the 50% isodose line, which is slightly higher than the 12 Gy currently prescribed to our patients with VS. After tolerating the procedure and undergoing a brief period of observation, the patient was discharged to her home.

One month following treatment, the patient had no complaints. The minimal discomfort from the pin placement for the Gamma Knife frame had resolved in a matter of days. She noticed no change in hearing, facial movement, facial sensation, or balance. The first follow up MRI was taken at 9.5 months, revealing a 17 mm lesion, which was concluded to be a reduction in size upon direct comparison with the preoperative MRI. At 10 months there was no change in her symptoms, other than episodes of pain and popping, which was perceived in both ears. Imaging at 22 months revealed further decrease in all tumour dimensions, with no change in symptoms.

**Figure 2:**
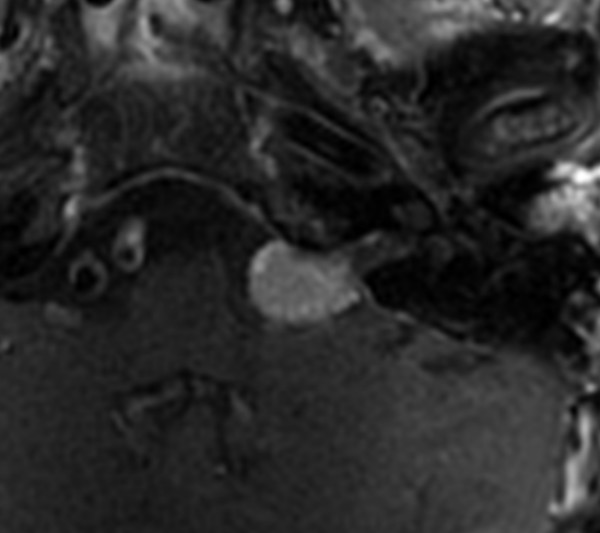
**Post-treatment MRI**. Most recent enhanced T1-axial MRI of the brain showing an enhancing lesion of the left cerebellopontine angle with a smaller maximum diameter than prior to gamma knife treatment.

The patient's most recent follow up occurred 44.5 months following her Gamma Knife procedure. At that time, MRI showed a 15 mm tumour, which was visibly smaller that in the study two years prior (see Figure 2). The patient did not comply with requests to obtain a follow up audiogram; however, she described no decrease in her hearing function. Symptoms of vertigo persisted, and she experienced a single brief episode of pain above the left auricle. Otherwise, there was no evidence for progressive cranial nerve disease or other complications, such as hydrocephalus or secondary malignancy. Overall, the tumour exhibited a stable appearance and reduction in size based on linear measurements. In addition to tumour control, her symptoms had not progressed, and therefore her Gamma Knife treatment was regarded as successful.

### Review of Relevant Literature

The recommended treatment for acoustic neuroma may depend on a number of factors; the tumour size, symptoms, patient age, and life expectancy. In practice, patients primarily choose observation, microsurgery, or radiosurgery, and the discipline of the attending physician is the greatest predictor of treatment choice[4,10]. This identifies the need for stronger evidence-based guidelines to reduce physician bias, necessitating more thorough comparison of the available interventions[9]. With all modalities, the primary endpoint sought is tumour control. This is generally defined as prevention of additional growth. Secondary treatment goals include alleviating symptoms and minimizing complications. Traditionally, the predominant measure of such outcomes has been the preservation of hearing and facial nerve function; however, there is a desire to evaluate other measures of patients' experience and quality of life, including the symptoms of tinnitus, dizziness, and headache[4]. This would provide patients a more complete picture to utilize in choosing a treatment strategy.

On average, vestibular schwannomas are slow growing; therefore, conservative management by periodic MRI is reasonable. In two large meta-analyses, the mean growth rate of tumours undergoing observation was determined to be 1.9 mm per year[5,8]. Unfortunately, the behaviour of an individual tumour is difficult to predict. Some may shrink spontaneously, while others may grow at rates 10 times that of the average tumour[11]. Growth rates are not always constant, and even large data sets identify few predictive variables for future growth[8]. The only indicators to this point are previous tumour growth, extracanalicular growth, and a younger age. A statistically significant (p < 0.01) difference in growth rates for intracanalicular and small tumours, compared to larger tumours of the cerebellopontine angle (CPA), was identified in two studies [3,12]. Larger tumours are more likely to grow and also grow faster. This intuitive result may be attributed to a greater number of proliferating cells, increased vascular perfusion, and greater spatial freedom for tumours of the CPA. The difficulty in predicting growth of VS is a risk patients undergoing observation must take into consideration.

The outcome for patients managed conservatively has been the subject of several reviews. *Yamakami et al. *reported that over a mean follow-up of 3.1 years, 51% of tumours grew, 20% eventually required intervention, and 37% of patients ultimately lost useful hearing (N = 903 patients)[5]. In a meta-analysis by *Smouha et al.*, 43% showed growth, 20% required treatment, and 51% of patients experienced hearing loss over a mean follow-up of 3.2 years (N = 1345 patients)[8]. *Myrseth et al. *reviewed the literature and determined that over a 3 year period 30-50% of patients undergoing conservative management will lose useful hearing and 15-50% will ultimately require active treatment[4]. Patients must be aware that a significant fraction managed conservatively will eventually require intervention or experience progression of symptoms.

The decision to undergo conservative management is not straightforward, although some treatment guidelines have been proposed. An algorithm designed by *Smouha et al. *recommends observation in elderly patients, with small tumours, and no symptoms other than hearing loss[8]. They define elderly patients as over 45 years and small tumours as less than 25 mm in diameter. Upon observing growth beyond 2 mm/year or changes in symptoms, intervention is indicated. Unfortunately there is little correlation between symptoms and tumour size, and patient quality of life may diminish even without growth[4]. Therefore, treatment may also be indicated when symptoms progress but the tumour size remains static. Concerns about patient compliance have also been voiced, and high attrition rates were noticed in some conservative management studies[8]. Nonetheless, observation remains a practical choice for many patients.

Historically, surgical removal has been the standard for treating vestibular schwannomas. Surgical resection of a VS was first documented in 1894[4]. Early on, mortality was above 50% until modern practices reduced that number to below 1%[9]. Today, surgery remains complicated, but the surgical microscope and improved techniques offer excellent tumour control with significantly reduced morbidity and mortality[4,5]. *Kaylie et al. *reviewed data about surgical outcomes from the 1990's, including 2579 patients who underwent surgical removal of VS. Tumour control, measured as lack of recurrence, was achieved in greater than 98% of cases[13]. In a similar analysis, *Yamakami et al. *reported 96% of tumours were completely removed, and only 1.8% recurred, following microsurgery in 5005 patients[5]. For larger tumours, the efficacy of microsurgery is unmatched, and *Nikolopoulos et al. *report that for tumours larger than 3 cm "it would be unethical to treat other than by surgical removal."[9].

Concerns about microsurgical intervention stem from the potential morbidities associated with intracranial procedures. Tumour size and surgeon's experience both play a role in the risks to the patient[4]. Hearing loss is an obvious morbidity, and deafness reportedly occurs in 50% of patients, although long term follow-up data is lacking[8]. Hearing preservation has been reported at very low rates with tumour sizes greater than 20 mm[4]. Meta-analysis of the literature from the 1990's identified other complications in 21.9% of cases[13]. The mortality rate was very low (0.3%) and CSF leak was the most common morbidity (10.95%). Other complications included facial nerve transection (3.9%), meningitis (1.2%), and damage to other cranial nerves (1.1%). A more recent review of the literature reports mortality in 0-2%, CSF leakage in 3-15%, facial nerve transection in 2.5-7%, and meningitis in 1-3%[4]. Data is limited on the impact of surgery on tinnitus, vertigo, and balance. Despite improved techniques, significant risks do still exist with surgery. Some patients may desire less invasive intervention while others may not be surgical candidates at all because of comorbid conditions.

Stereotactic radiosurgery (SRS) is an accepted alternative to microsurgery for smaller tumours and non-surgical candidates, offering similar tumour control rates[4,5,14,15]. Meta analysis including studies from the 1990's identified an average tumour control rate of 91% with Gamma Knife radiosurgery[13]. A more recent review identified a range of control rates, from 89-100% reported in various studies. Complicating the interpretation of such results, many of which report on patients first treated over a decade ago, is that Gamma Knife technology and treatment planning continues to undergo significant evolution. Improvements in imaging resolution and computer planning software, have allowed physicians to better spare adjacent brainstem and nerve structures[16,17]. Furthermore, the therapeutic dose used for treating VS has decreased over the past two decades, with marginal doses of 12 Gy and maximum doses of 20-25 Gy identified as current standards[4]. Therefore, controlling vestibular schwannomas with fewer risks may be accomplished with contemporary SRS.

Patients treated with SRS can have a similar complication profile to those treated with intracranial surgery. Meta-analysis from early experience showed that 44% with serviceable hearing prior to treatment retained their ability after SRS, a statistically equivalent rate to the surgical data[13]. This evidence also suggest that 37.9% of patients have other complications[13]. Trigeminal neuropathy was experienced by 36%, 6% had facial nerve injury, and 1.9% developed hydrocephaly. Hearing loss following radiosurgery has been linked to dose margins overlapping the cochlea, or brainstem nuclei of the cochlear nerve[16,18]. Several studies indicate that lower doses and more precise planning software used currently, offer greater preservation of hearing and reduction of facial neuropathy compared to surgery[4,5,14,15,19]. This was indicated in a recent review where hearing preservation rates range from 50-89% in more contemporary studies[4]. The rates of other complications were 3-5% for trigeminal neuropathy, 1-4% for facial neuropathy, and 2-4% for hydrocephaly[4]. With radiation exposure, there also remains a small risk of developing secondary neoplasm, but few studies of VS include follow-up times necessary to determine the true risks. Ultimately, the side effect profile for radiosurgery appears to be comparable to or slightly better than microsurgery.

Fractionated stereotactic radiotherapy (SRT) is an alternative use of radiation in the treatment of vestibular schwannoma. A review of available evidence suggests that SRT may offer even better hearing preservation and lower cranial nerve toxicity than SRS based on biologic models[20]. This includes similar tumour control rates with hearing preservation greater than 90%. Unfortunately, being a newer therapy, there are not yet long-term comparative outcome studies to evaluate the relative efficacy of SRT compared to SRS or microsurgery [16].

## Conclusions

We report a case where a vestibular schwannoma presented without hearing loss. Rather, the patient experienced dizziness and an audible "clicking" sound, an unusual symptom profile. A causal relationship between vestibular schwannoma and "clicking" sensations cannot be concluded, however, physicians should be aware of a potential correlation. We advocate considering vestibular schwannoma as part of the differential diagnosis in any case where central neurological symptoms correlate with hearing problems, including sensations of "clicking" or "popping" that might otherwise be attributed to extracranial problems like eustachian tube defect.

In the case reported, Gamma Knife radiosurgery, utilizing a marginal dose of 13 Gy and contemporary planning techniques, resulted in tumour control and hearing preservation after 44.5 months of follow-up. In general, we lack a long-term understanding of the benefits provided by such treatment protocols. We continue to evaluate the advantages of including lower dose therapy, high-resolution stereotactic magnetic resonance, fractionation, and improved precision of treatment-planning software. However, the best available evidence continues to support stereotactic radiosurgery as a viable intervention with tumour control rates above 90% and improved risks of complication relative to surgery. In patients with functional hearing prior to treatment, modern stereotactic radiosurgery offers greater potential for preserved function, at rates above 50%.

Ultimately, each patient's tumour, symptoms, and therapeutic goals are unique, requiring a customized therapeutic plan. Physicians should be aware of the full breadth of presentations associated with vestibular schwannomas, including auditory changes other than sensory loss or tinnitus. We also advocate maximizing informed decision-making, by encourage patients to meet with a radiation oncologist, neurosurgeon, and neuro-otologist prior to treatment. In this way patients can best weigh the strong efficacy of surgery with the low risk of observation, while evaluating radiosurgery as an alternative somewhere in between. We hope this review will aid physicians in the diagnosis and management of patients with vestibular schwannomas.

## Consent

Written informed consent was obtained from the patient for publication of this case report.

## Competing interests

The authors declare that they have no competing interests.

## Authors' contributions

BJA reviewed relevant clinical data for this case report, reviewed the current literature, and drafted the manuscript. CML and WTL provided clinical expertise and participated in drafting the manuscript. NAG and ARM provided clinical expertise relevant to the case report. All authors read and approved the final manuscript.
